# Epidemiological characteristics of the first 53 laboratory-confirmed cases of COVID-19 epidemic in Hong Kong, 13 February 2020

**DOI:** 10.2807/1560-7917.ES.2020.25.16.2000155

**Published:** 2020-04-23

**Authors:** Kin On Kwok, Valerie Wing Yu Wong, Wan In Wei, Samuel Yeung Shan Wong, Julian Wei-Tze Tang

**Affiliations:** 1 JC School of Public Health and Primary Care, The Chinese University of Hong Kong, Hong Kong Special Administrative Region, China; 2 Stanley Ho Centre for Emerging Infectious Diseases, The Chinese University of Hong Kong, Hong Kong Special Administrative Region, China; 3 Shenzhen Research Institute of The Chinese University of Hong Kong, Shenzhen, China; 4 Respiratory Sciences, University of Leicester, Leicester, United Kingdom

**Keywords:** SARS-CoV-2, COVID-19, outbreak, epidemiology, social distancing, containment delay, serial interval, secondary attack rate

## Abstract

**Background:**

COVID-19, caused by SARS-CoV-2, first appeared in China and subsequently developed into an ongoing epidemic. Understanding epidemiological factors characterising the transmission dynamics of this disease is of fundamental importance.

**Aims:**

This study aimed to describe key epidemiological parameters of COVID-19 in Hong Kong.

**Methods:**

We extracted data of confirmed COVID-19 cases and their close contacts from the publicly available information released by the Hong Kong Centre for Health Protection. We used doubly interval censored likelihood to estimate containment delay and serial interval, by fitting gamma, lognormal and Weibull distributions to respective empirical values using Bayesian framework with right truncation. A generalised linear regression model was employed to identify factors associated with containment delay. Secondary attack rate was also estimated.

**Results:**

The empirical containment delay was 6.39 days; whereas after adjusting for right truncation with the best-fit Weibull distribution, it was 10.4 days (95% CrI: 7.15 to 19.81). Containment delay increased significantly over time. Local source of infection and number of doctor consultations before isolation were associated with longer containment delay. The empirical serial interval was 4.58–6.06 days; whereas the best-fit lognormal distribution to 26 certain-and-probable infector–infectee paired data gave an estimate of 4.77 days (95% CrI: 3.47 to 6.90) with right-truncation. The secondary attack rate among close contacts was 11.7%.

**Conclusion:**

With a considerable containment delay and short serial interval, contact-tracing effectiveness may not be optimised to halt the transmission with rapid generations replacement. Our study highlights the transmission risk of social interaction and pivotal role of physical distancing in suppressing the epidemic.

## Introduction

Severe acute respiratory syndrome coronavirus 2 (SARS-CoV-2), which is responsible for coronavirus disease (COVID-19), first appeared in Wuhan, China, in early December 2019, where it caused an epidemic which subsequently spread to other countries. Following a rising number of confirmed cases and evidence of human-to-human transmission [1], the World Health Organization declared COVID-19 a Public Health Emergency of International Concern on 30 January 2020 [2] and a pandemic on 11 March 2020 [3]. As at 13 February 2020, there were 46,997 confirmed cases among 25 countries and 1,369 related deaths [4].

After the first confirmed COVID-19 case was imported to Hong Kong on 22 January 2020 [5,6], the government promptly introduced multi-pronged interventions to suppress the spread of SARS-CoV-2 [7]. Such interventions included physical distancing (school closures, work-from-home arrangements for civil servants, suspension of public leisure and recreational facilities) and border restriction. Contact tracing followed by quarantine and screening was also carried out when cases were identified, however the initially mild clinical presentation of COVID-19 can hamper its diagnosis [8].

With an average daily 12.5 contacts per individual in Hong Kong [9], it is essential to assess the transmission risk of COVID-19 posed to close contacts of cases. In fact, for previous outbreaks caused by other coronaviruses, such transmission risk is documented. During the 2003 severe acute respiratory syndrome (SARS) outbreak in Hong Kong, 16.1% probable cases were attributable to household transmission [10]. Three generations of secondary infections, featured by a history of direct contacts, were also observed in the 2015 Middle East respiratory syndrome (MERS) outbreak in South Korea [11]. To this end, with the first 53 cases in Hong Kong, this study addresses four epidemiological aspects of COVID-19: (i) quantifying delay in isolating confirmed cases from symptom onset (thereafter denoted as ‘containment delay’); (ii) exploring factors associated with the containment delay; (iii) estimating the clinical-onset serial interval (thereafter denoted as ‘serial interval’); and (iv) estimating the secondary attack rate.

## Method

### Data retrieval

We extracted data of confirmed COVID-19 cases and corresponding close contacts from the publicly available information released by the Hong Kong Centre for Health Protection (CHP) [12,13]. The cut-off date for data extraction was 13 February 2020. We retrieved cases’ characteristics, including case identifier, demographical information (age, sex, presence of comorbidities), potential source of infection, travel history, number of doctor consultations between symptom onset and isolation (thereafter denoted as ‘number of doctor consultations’), number of close contacts, epidemiological links among cases, date of symptom onset and date of isolation. The case identifier used in this study was identical to that used by the CHP.

### Case, contact and cluster definitions

A laboratory-confirmed case (thereafter denoted as ‘a case’ or ‘a confirmed case’) referred to an individual who had nucleic acid of SARS-CoV-2 detected or had SARS-CoV-2 isolated in a clinical specimen [14]. Close contacts referred to anyone who: (i) provided care to the case (including a family member or healthcare worker) or had other close physical contact; or (ii) stayed at the same place (including household members or visitors) while the case was ill [14]. A cluster was defined as two or more confirmed cases with epidemiological links based on disease characteristics and contact patterns [15]. An index case referred to the case with the earliest symptom onset in a cluster [16]. A secondary case referred to the first generation of infection induced by an index case following contact with this case, whereas infection induced by a secondary case was the second generation of infection [11].

### Definitions of epidemiological parameters

**Figure 1:**
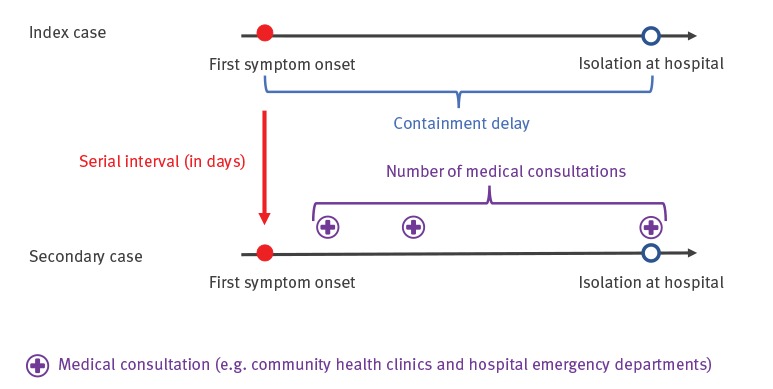
Chronological timeline of primary and secondary cases before isolation, Hong Kong, 2020

COVID-19: coronavirus disease; SARS-CoV-2: severe acute respiratory syndrome coronavirus 2.

Containment delay referred to the failure to initiate any form of physical isolation after the first symptom onset, and was defined as the time interval between date of symptom onset and date of isolation (Figure 1). Serial interval, was the time elapsed between symptom onsets of two successive generations of cases (Figure 1) [16,17]. Secondary attack rate referred to the probability that infection occurred among susceptible individuals in a cluster [18].

The arrows represent timelines. Three cases of 53 were hospitalised for other reasons than COVID-19 (range of hospital stay: 3 to 8 days), but were subsequently found to be infected by SARS-CoV-2 and were isolated.

### Statistical analysis

We summarised the characteristics of confirmed cases with descriptive statistics such as mean, median, range, standard deviation (SD) and frequency, 95% bootstrapped confidence interval (bCI) and 95% binomial confidence interval (binCI). A generalised linear regression model was adopted to identify factors associated with containment delay. A statistical significance based on p value of 0.05 was set.

A doubly interval-censored likelihood function was defined to fit three distributions (gamma, lognormal, Weibull) to empirical containment delays (Supplement S1) and serial intervals (Supplement S2) with right truncation, as in previous studies [19,20]. Estimation for these two time intervals was conducted in a Bayesian framework, and 95% credible intervals (CrI) were computed. Fitting of distributions was compared by widely applicable information criteria (WAIC). For serial intervals, the estimation was based on infector–infectee paired data (thereafter denoted as ‘paired data’). The secondary attack rate was calculated by dividing the number of confirmed cases in the first generation by the total number of close contacts. All analyses were conducted in R (v3.6.3).

### Ethical statement

This study was conducted using publicly available data released by the Hong Kong CHP; therefore, no ethical approval was needed.

## Results

### Characteristics of cases

As at 13 February 2020, there were 53 cases reported by the CHP. Table 1 and Table S1 detail their baseline characteristics. The majority were male (29/53), and the mean age of the overall cases was 55.7 years (range: 22–91 years). Eleven cases had comorbidities, and 40 cases had sought doctor consultations from community health clinics or hospital emergency departments before being diagnosed. The mean number of doctor consultations was 2.07 (95% bCI: 1.71 to 2.45).

**Table 1:** Characteristics of the first 53 laboratory-confirmed cases of coronavirus disease reported in Hong Kong, 23 January–13 February 2020^a^

Characteristics	Number of cases (n = 53)
**Sex**	
Male	29
Female	24
**Age group in years**	
22–39	12
40–69	31
≥ 70	10
**Presence of comorbidity**	
Yes	11
No	28
Missing	14
**Potential source of infection**	
Imported	13
Local	
Unknown source or possible local transmission	15
Close contacts of imported case	2
Close contacts of local and possible local case	23

^a^ The date range from 23 January to 13 February corresponds to the period when the 53 cases were reported.

**Figure 2:**
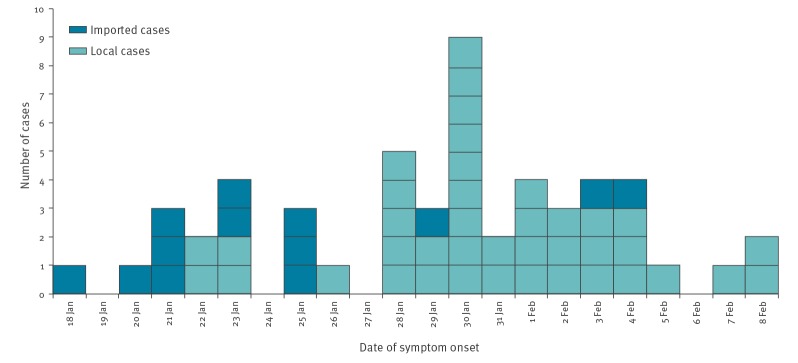
Distribution of local and imported laboratory-confirmed cases of coronavirus disease in Hong Kong by date of symptom onset, 18 January–8 February 2020^a^ (n = 53 cases)

^a^ The date range from 18 January to 8 February corresponds to the period when the 53 cases presented symptom onset.

Concerning the source of infection, 13 cases were imported (thereafter denoted as ‘imported cases’); whereas 40 cases had a possible local source (thereafter denoted as ‘local cases’), which could further be classified as: (i) unknown source or possible local transmission (15/40); (ii) close contacts of imported cases (2/40); and (iii) close contacts of local cases and/or possible local cases (23/40). Two imported cases were intercepted at borders (cases 1 and 7), and two other cases, including one imported and one who was a close contact of an imported case, were under quarantine during symptom onset (cases 8 and 15). The distribution of cases (Figure 2) revealed three turning points in the transmission dynamics: cases were mostly imported in late January 2020, followed by a surge of local cases with unknown sources, and finally there was a substantial reduction of imported cases in early February 2020.

### Containment delay

After excluding four cases who did not contribute to COVID-19 transmission in the Hong Kong community (cases 1, 7, 8 and 15), data of 49 cases were used to quantify the containment delay (Supplement S1).

The overall mean containment delay was 6.39 days (95% bCI: 5.37 to 7.45) with a SD of 3.87 days (95% bCI: 3.36 to 4.31) (Table S2). The containment delays for imported cases and local cases were 1.70 days (95% bCI: 0.90 to 2.60) and 7.58 days (95% bCI: 6.54 to 8.62) respectively.

**Figure 3:**
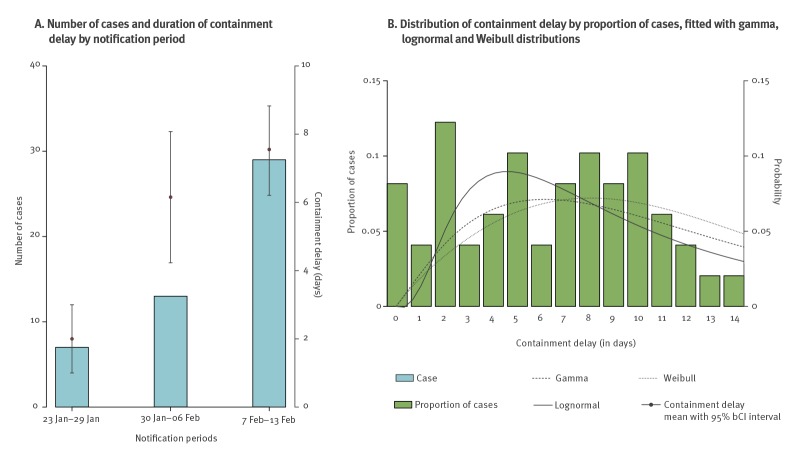
Characterisation of the duration of containment delay relative to (A) outbreak period and numbers of cases and (B) proportion of cases, Hong Kong, 23 January–13 February 2020 (n = 49 cases)^a^

A temporal increase in containment delay was observed between the following successive periods (Figure 3A): (i) from 23 January 2020 to 29 January 2020 (mean: 2.00 days; 95% bCI: 1.00 to 3.00); (ii) from 30 January 2020 to 6 February 2020 (mean: 6.15 days; 95% bCI: 4.23 to 8.08); and (iii) from 7 February 2020 to 13 February 2020 (mean: 7.55 days; 95% bCI: 6.21 to 8.83).

bCI: bootstrapped confidence interval.

^a^ Four of the 53 cases in the current study who did not contribute to coronavirus disease transmission in the Hong Kong community are excluded for the containment delay characterisation in this Figure, leaving a total of 49 cases. The date range from 23 January to 13 February corresponds to the period when the 49 cases were reported.

The Weibull distribution fitted the empirical containment delay best (i.e. with the lowest WAIC of 383.9 vs 385.1 and 389 for the gamma and lognormal distributions respectively) (Table S2), and was assumed in the reporting of this section unless specified. Accounting for right truncation, the estimated mean and SD of containment delay were respectively 10.38 days (95% CrI: 7.15 to 19.81) and 5.97 days (95% CrI: 3.23 to 13.75) (Figure 3B). Without adjusting for right truncation, a shorter containment delay (mean: 7.04 days; 95% CrI: 5.89 to 8.25) with a smaller SD (3.53 days; 95% CrI: 2.86 to 4.45) was observed (Table S2).

The estimates from gamma and lognormal distribution are shown in Table S2.

### Factors associated with containment delay

Age and sex were adjusted for in the multivariate regression analysis. In line with earlier findings (Figure 3A), we identified a significant time trend in containment delay (Table 2, Model 1): (i) from 30 January 2020 to 6 February 2020 (regression coefficient (RC): 4.61 days; 95% confidence interval (CI): 1.42 to 7.81); (ii) from 7 February 2020 to 13 February 2020 (RC: 6.05 days; 95% CI: 3.21 to 8.90). Compared with local cases, imported cases experienced shorter containment delay by 6.08 days (95% CI: −8.26 to −3.91) (Table 2, Model 2); whereas containment delay, with further adjustment for presence of comorbidities, was lengthened by 2.08 days (95% CI: 1.16 to 2.99) per doctor consultation (Table 2, Model 3).

**Table 2:** Factors associated with containment delay, Hong Kong, 23 January–13 February 2020 (n = 49 cases)^a^

Factors	Regression coefficient (95% confidence interval)		
	Model 1 (n = 49)^a^	Model 2 (n = 49)^a^	Model 3 (n = 35)^a,b^
**Age group in years**			
22–39	Reference	Reference	Reference
40–69	1.51 days (−0.90 to 3.93)	1.69 days (−0.47 to 3.86)	−0.57 days (−3.26 to 2.13)
≥ 70	2.54 days (−0.49 to 5.58)	2.18 days (−0.53 to 4.88)	4.08 days (−0.05 to 8.21)
**Sex**			
Female	Reference	Reference	Reference
Male	−1.31 days (−3.25 to 0.62)	−0.44 days (−2.21 to 1.33)	−3.16 days (−5.39 to −0.92)
**Case notification period start and end in day/month **			
23/01 to 29/01	Reference	Not applicable	Not applicable
30/01 to 06/02	4.61 days (1.42 to 7.81)	Not applicable	Not applicable
07/02 to 13/02	6.05 days (3.21 to 8.90)	Not applicable	Not applicable
**Potential source of infection**			
Local	Not applicable	Reference	Not applicable
Imported	Not applicable	−6.08 days (−8.26 to −3.91)	Not applicable
**Number of doctor consultations^c^ before isolation**			
Number of doctor consultations^c^ before isolation	Not applicable	Not applicable	2.08 days (1.16 to 2.99)
**Presence of comorbidities**			
No	Not applicable	Not applicable	Reference
Yes	Not applicable	Not applicable	1.52 days (−1.06 to 4.10)

^a^ Cases intercepted at borders (cases 1 and 7) or quarantined during symptom onset (cases 8 and 15) were excluded.

^b^ Cases with missing number of doctor consultations before isolation (cases 30–36) or missing comorbidity (cases 9-12, 29-36, 42 and 46) were excluded.

^c^ From community health clinics or hospital emergency departments.

The date range covered by the table, from 23 January to 13 February, corresponds to the period when the 49 cases were reported. Model 1 explores the association of the containment delay and different periods of the outbreak after adjusting for age and sex. Model 2 explores the association between the containment delay and the potential source of infection after adjusting for age and sex. Model 3 explores the association between the containment delay and the number of doctor consultations before isolation after adjusting for age, sex and presence of comorbidities.

### Serial interval

Serial intervals were estimated from 26 (probable: 9; certain: 17) paired data (Supplement S2). The mean serial interval estimated from all 26 paired data was 4.58 days (95% bCI: 3.35 to 5.85), with a SD of 3.28 days (95% bCI: 2.18 to 4.01).

The lognormal distribution fitted the empirical serial interval best (WAIC: 214.7 vs 217.6 and 219.0 for gamma and Weibull respectively; Figure S1; Table S2), and was assumed in the reporting in this section unless specified. Adjusting for right truncation, the mean serial interval was 4.77 days (95% CrI: 3.47 to 6.90), with a SD of 4.08 days (95% CrI: 2.26 to 8.05); whereas without adjusting for right truncation, the mean serial interval was 4.41 days (95% CrI: 2.46 to 11.15), with a SD of 3.44 days (95% CrI: 2.13 to 5.93).

Restricting our analysis to 17 certain paired data resulted in longer serial intervals (empirical: 6.06 days; estimated, truncated: 6.23 days; estimated, non-truncated: 5.86 days) (Table S2). The estimates from gamma and lognormal distribution are given in Table S2.

### Secondary attack rate

**Figure 4:**
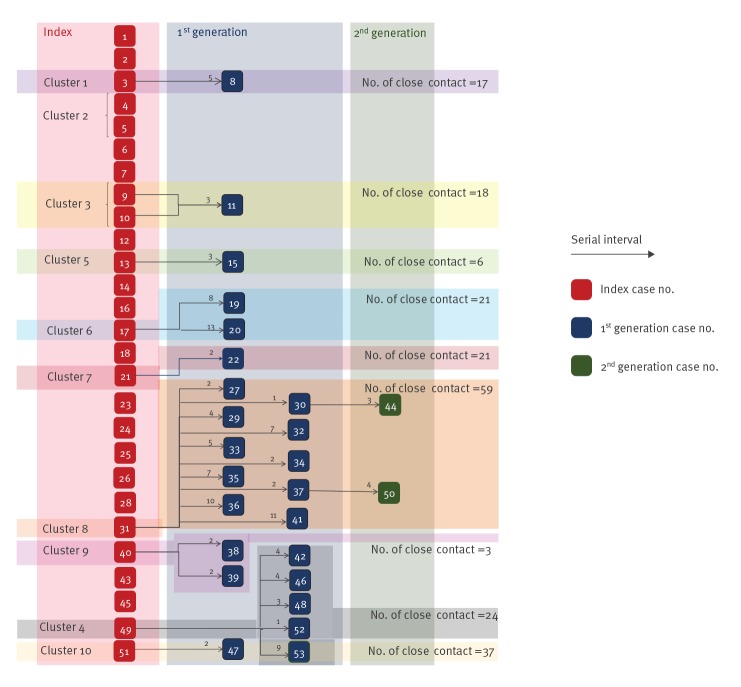
Transmission chains by generation, Hong Kong, 23 January–13 February 2020 (n = 53 cases)^a^

We illustrated the transmission chains of all index cases and their subsequent generations of infections (Figure 4; Figure S2). There were 10 clusters (Figure S2) with a mean size of 3.8, and a maximum size of 13. Most of the earliest clusters (Clusters 1, 2, 3, 4 and 7) were linked to close contact of cases with travel history in China. Among the 206 close contacts of cases who had been quarantined or isolated by the CHP, 24 became cases in the first generation of infection. Therefore, the estimated secondary attack rate was 11.7% (95% binCI: 7.61 to 16.8).

No.: number; SI: serial interval in days.

Cases 3 and 8 were included in the calculation of secondary attack rate. Although they both travelled to Wuhan during the incubation period, Case 8 developed symptoms, during quarantine in Hong Kong, 6 days after Case 3.

Cases 4 and 5 (Cluster 2) were excluded in the secondary attack rate calculation since they had the same travel history (to Wuhan) and the same date of symptom onset.

Cases 21 and 22 were included in the analyses of SI and secondary attack rate. While they had the same travel history (to China), the dates of symptom onset differed by 2 days.

## Discussion

With the first 53 cases occurring in the absence of drastic physical-level interventions, this study presents the ‘semi-intrinsic’ epidemiological properties of SARS-CoV-2 in Hong Kong. We report three important parameters: (i) the containment delay; (ii) the serial interval; and (iii) the secondary attack rate. For the estimation of the first two time intervals, the ongoing aspect of the epidemic was accounted for by right truncation of the data. We also identified factors associated with the containment delay.

The empirical containment delay was estimated as 6.39 days and could be as high as 10.38 days after adjusting for right truncation. When a delay in containment occurs for a case, this opens the opportunity for transmission of COVID-19 when symptoms are occurring. In this respect, although the differentiation between hospitalisation and isolation is sometimes blur in the literature, the onset-to-hospitalisation time can be considered as a proxy for the time when COVID-19 can spread during the symptomatic stage, which could theoretically be preventable. The reported duration of onset-to-hospitalisation in other studies ranged, on average, from 2 to 4 days [19,21,22] and could be up to 10 days [23].

The estimates of the containment delay in Hong Kong suggest that it was longer than in other places outside Wuhan, such as Shenzhen, China (4.6 days) [24] and Singapore (5.6 days) [25]. Moreover, in Hong Kong the containment delay increased over time whereas it declined considerably in Singapore [25]. Factors affecting the containment delay in Hong Kong were found to be the source of infection (with shorter delays for imported cases than local ones) and the number of doctor consultations before isolation. The progressive increase of the containment delay appeared to reflect the varying transmission dynamics of COVID-19. Most early cases in Hong Kong were imported from other places in China, such that recent travel (in China) was initially considered a risk factor of infection. As the epidemic progressed and restrictions at the border were put in place, travel history could no longer be a criterion for diagnosis. The absence of rapid diagnostic tools, together with the non-specific symptoms of COVID-19, which can overlap with those of other respiratory illnesses that were occurring during the winter influenza season, jointly increased the difficulty to discern SARS-CoV-2 infections. This also explains the positive association between containment delay and the number of doctor consultations before isolation.

The serial interval, considering certain and probable paired data together, was empirically estimated as 4.58 days, and, after right truncation adjustment, as 4.77 days. Our findings were similar to those of other work by Nishiura and colleagues (4.0–4.6 days) [20], and Du and colleagues (3.96 days) [26]. Nevertheless, a longer serial interval was reported by Li and colleagues (mean: 7.5 days; 95% CI: 5.3–19.0) [27] and Bi and colleagues (6.3 days) [24]. It should be noted however, that in the current study, data certainty considerably affected the serial interval estimates (which differed by 1.5 days depending if both certain and probable paired data were used, or if only certain paired data were used). This suggests that clear information on epidemiological links is needed to unravel this important parameter.

For the secondary attack rate, our estimate (ca 10%) among close contacts was lower than that reported by Liu and colleagues (35%) [18], and Bi and colleagues (15% among household contacts) [24]. The comparatively low secondary attack rate in Hong Kong might be attributable to the high level of civil engagement in individual-level preventive measures [28].

Our study has several public health implications. First the vague differences between COVID-19 and other co-circulating respiratory diseases, which may have impacted on the containment delay, suggest the need for active screening of COVID-19. On 19 February 2020, the Hong Kong government implemented the Enhanced Laboratory Surveillance Programme to collect deep-throat specimens from outpatients for active diagnosis [29]. The extent to which this programme reduced containment delay remains to be quantified. Nevertheless, despite its unclear immediate benefit, this heightened surveillance network could collect data of long-term investigational value.

Second, the short serial interval (4.77 days) of COVID-19 can impede contact tracing. As cases were generated quickly through transmission chains, health officials had to race against time to trace contacts. The serial interval also appeared to be somewhat shorter than the reported incubation period for COVID-19 (which is on average 5–6 days [27,30,31], and can be up to 14 days [19]), suggesting that pre-symptomatic transmission could have occurred (Figure S3). This finding was in line with the results of work by Du and colleagues [26], who delineated that among reports of transmission between infector–infectee, 12.6%  were due to pre-symptomatic transmission. The short serial interval, combined with pre-symptomatic transmission, containment delay (which fosters symptomatic transmission) and high secondary attack rate (which is almost double than that of SARS (6.3%) [32]) suggests that collective and drastic physical-distancing policies, such as limiting the size of people gatherings, issuing stay-at-home order, mass closure of public venues or even city lockdown may be needed to contain the COVID-19 epidemic. Should these non-pharmaceutical interventions not work, we might need to resort to cautiously enhancing population herd immunity [33].

Third, tight infection control should be imposed on healthcare and long-term care facility settings. Pre-symptomatic transmission [34] and containment delay (as identified in the current study) eased the penetration of COVID-19 into long-term care facilities (LTCFs) in the United States. Elderly persons with comorbidities have been shown to be vulnerable to COVID-19 and to be likely to experience severe outcomes [35]. The identification in March 2020 of COVID-19 in 30 LTCFs in the United States [36] as well as the report of a considerable number of deaths in elderly homes in Italy [37] point to the potential for imminent outbreaks in these settings. Besides LTCFs, based on past experience from nosocomial outbreaks caused by other coronaviruses, such as the ones responsible for SARS in Hong Kong in 2003 [38] and MERS in South Korea in 2015 [11], attention should be paid to inpatients hospitalised for other illnesses than COVID-19, who might be in the pre-symptomatic stage of SARS-CoV-2 infection. Moreover, keeping alert to patients who might have symptoms not yet recognised as being due to COVID-19 may also be important. Kraemer et al. reported that from the onset of COVID-19, the time to confirmation (onset-to-confirmation time: 6.17 days) was longer than the time to hospitalisation (onset-to-hospitalisation time: 2.96 days) [22], suggesting that outbreaks of COVID-19 in hospitals were possible. In fact, among the first 53 cases in Hong Kong, there were three cases (cases 12, 17, and 29) hospitalised for illnesses other than COVID-19 who turned out to be confirmed cases of COVID-19 as well. In addition to LTCFs and hospitals, the positive association between number of doctor consultations and containment delay in our study pinpoints the exposure of outpatient settings to COVID-19. Therefore, guideline on patient flow management for outpatient settings would reduce the cross infections that can arise from their packed environments.

Fourth, this study offers useful information to the scientific community and policymakers, particularly input for mathematical forecasting models. It presents several aspects of COVID-19 epidemiology and tries to link them up to profile the course of the epidemic. The intrinsic epidemiological properties captured in this study can be of reference value to countries where drastic interventions have not yet been implemented. It can also serve as the baseline to evaluate the effectiveness of interventions (for example, whether the epidemiology of COVID-19 is altered after interventions, which would probably result in a lower secondary attack rate).

This study has nevertheless four limitations. First, the recall bias of cases might have affected data accuracy, including their self-reported symptom onset date and contact history. Second, unclear data reporting, such as those of the social contact network in a cluster, led to subsequent data assumptions. Third, insufficient information release during the outbreak limited the epidemiological exploration of this study. Fourth, the limited understanding on clinical characteristics of COVID-19 obscure the definition of symptom onset, which could be characterised by respiratory or systemic symptoms (such as fever).

To conclude, this study outlined the intrinsic epidemiological characteristics of SARS-CoV-2. Our estimates were comparable with those documented by others. Variability of the parameters, which govern the transmission dynamics of COVID-19, should be considered for future interpretation. From the results, it appears that pre-symptomatic transmission and containment delay, which in turn fosters symptomatic transmission, occurred during the early phase of the COVID-19 epidemic in Hong Kong. Based on the results, control strategies were designed. Considering the non-specific symptoms of COVID-19 and the contagious nature of the responsible virus, sustainable physical distancing is recommended. 

## Supplementary Data

590000 Supplement
