# Supplementary material for: Epidemiological characteristics of the first 53 laboratory-confirmed cases of COVID-19 epidemic in Hong Kong, 13 February 2020
Source: Euro Surveill. 2020 Apr 23;25(16):2000155. doi: 10.2807/1560-7917.ES.2020.25.16.2000155 (PMC7189647; doi:10.2807/1560-7917.ES.2020.25.16.2000155)
Supplement: Supplement [file 2000155_KWOK_Supplement.pdf]

ONLINE SUPPLEMENTARY FILE

"This supplementary material is hosted by Eurosurveillance as supporting information alongside the article [Epidemiological characteristics of the first 53 laboratory-confirmed cases of COVID-19 epidemic in Hong Kong], on behalf of the authors, who remain responsible for the accuracy and appropriateness of the content. The same standards for ethics, copyright, attributions and permissions as for the article apply. Supplements are not edited by Eurosurveillance and the journal is not responsible for the maintenance of any links or email addresses provided therein."

Supplement S1. Estimation of containment delay

Case data construction

A complete dataset with report dates and onset dates of 53 confirmed cases was extracted from the Hong Kong Centre for Health Protection (CHP) website. Cases intercepted at the border checkpoints (cases 1 and 7), and those quarantined during their symptom onset (cases 8 and 15) were excluded from analysis. To estimate the containment delay, we further extracted isolation date of confirmed cases from documents of CHP press conference. A total of 25 confirmed cases with certain isolation dates were extracted. We further assumed each of the 9 additional isolation dates to be the same day as its corresponding confirmation dates because the precise isolation date was not given. We imputed the rest 15 cases' isolation dates by the formula: [report date – (mean of isolation to report date in that report date)]. The data of 53 cases is available online: https://github.com/kkokwok/eurosurvellience

Among imported cases, three cases (cases 2, 6, and 25) had symptom onset 1-2 days prior to arrival in Hong Kong, but we assumed that their onset dates were their corresponding arrival dates in Hong Kong.

Estimation model

As symptom onset data and isolation data were given in an exact date without time which resulted in discretized observed case to case intervals [1]. We assumed the symptom onset and isolation occurred equally likely any time during the day from 0:00 to 24:00 hours. We estimated the

containment delay by a Bayesian approach with doubly interval-censored likelihood function as follows:

$$L(\theta_g; D) = \prod_i \int_{A_{L,i}}^{A_{U,i}} \int_{B_{L,i}}^{B_{U,i}} g(a)h(b-a)dbda$$

where g(x) is the probability density function (PDF) of onset with uniform distribution, h(x) is the PDF of containment delay, D is the dataset for the estimation where onset and isolation were within the lower bounds and upper bounds (($A_{L,i}$, $A_{U,i}$) and ($B_{L,i}$, $B_{U,i}$)) respectively.

h(.) was fitted by three candidate distributions including Gamma, Lognormal and Weibull distribution.

We also take into account the selection bias during the ongoing epidemic with the right truncation using the derivative of h(x) with the following formula:

$$h'(b-a,a) = \frac{h(b-a)}{\int_0^{T-e} \frac{re^{(-ru)}}{1-e^{(-ru)}} H(T-a-u)du}$$

where T is report date of 53th case (13 February 2020), r is the exponential growth rate and H is the cumulative density function of h.

The model with the lowest widely applicable information criterion (WAIC) was selected as the best fitted model.

Supplement S2. Estimation of serial interval

With all available information from CHP and subjective assessment by authors on reporting certainty of data, we constructed two sets of infector-infectee onset times using (i) 26 certain and probable pairs; and (ii) 17 certain pairs to estimate the serial interval.

The same estimation model was also applied to estimation of serial interval where *i* represents the ith infector-infectee pair; ($A_{L,i}$, $A_{U,i}$) and ($B_{L,i}$, $B_{U,i}$) are the intervals for symptom onset of the infector and infectee respectively, and g(x) and h(x) are PDFs of exposure and serial interval respectively.

Table S1. Case-specific characteristics

Characteristics	Number of cases (N=53)	Case number
**Sex**		
Male	29	1, 2, 5, 6, 8, 10, 12, 13, 14, 17, 18, 21, 25, 26, 27, 28, 30, 32, 36, 38, 41, 43, 45, 46, 47, 49, 50, 51, 53
Female	24	3, 4, 7, 9, 11, 15, 16, 19, 20, 22, 23, 24, 29, 31, 33, 34, 35, 37, 39, 40, 42, 44, 48, 52
**Age**		
22-39	12	1, 11, 13, 18, 19, 27, 32, 34, 36, 48, 49, 53
40-69	31	2, 3, 4, 5, 6, 7, 8, 16, 17, 20, 21, 22, 23, 24, 25, 26, 30, 31, 33, 35, 37, 38, 39, 41, 42, 43, 44, 47, 50, 51, 52
≥70	10	9, 10, 12, 14, 15, 28, 29, 40, 45, 46
**Presence of comorbidities**		
Yes	11	8, 13, 15, 17, 24, 28, 38, 39, 43, 45, 47
No	28	1, 2, 3, 4, 5, 6, 7, 14, 16, 18, 19, 20, 21, 22, 23, 25, 26, 27, 37, 40, 41, 44, 48, 49, 50, 51, 52, 53
Missing	14	9, 10, 11, 12, 29, 30, 31, 32, 33, 34, 35, 36, 42, 46
**Source of infection**		
Imported	13	1, 2, 3, 4, 5, 6, 7, 8, 9, 10, 13, 25, 26
Local		
Unknown source or possible local transmission	15	12[a], 14[a], 16, 17, 18, 21[a], 23, 24, 27[a], 28, 40, 43, 45, 49, 51
Close contacts of imported case	2	11, 15
Close contacts of local and possible local case	23	19, 20, 22, 29, 30, 31, 32, 33, 34, 35, 36, 37, 38, 39, 41, 42, 44, 46, 47, 48, 50, 52, 53

[a] These cases were deemed as possible local cases because they had either travelled before the incubation period (case 12), or bypassed China for a short period (cases 14 and 21), or had contacted a possible case from China (case 27).

Table S2. Estimates of containment delay and serial interval (with 95% CrI) by three candidate distributions (gamma, log-normal, weibull) with truncated and non-truncated approaches and bootstrapping

Interval		Truncated			Non-truncated			Bootstrap
		Gamma	Log-normal	Weibull	Gamma	Log-normal	Weibull	
CD[a]	Mean	12.2 (7.76, 21.48)	11.25 (7.64, 17.95)	10.38 (7.15, 19.81)	7.08 (5.88, 8.49)	7.05 (5.80, 8.68)	7.04 (5.89, 8.25)	6.39 (5.37, 7.45)
	SD	8.49 (4.44, 17.02)	10.53 (5.38, 21.65)	5.97 (3.23, 13.75)	4.22 (3.21, 5.77)	5.1 (3.60, 7.49)	3.53 (2.86, 4.45)	3.87 (3.36, 4.31)
	WAIC	385.1	389	383.9	247.3	254.2	243	NA
SI[b]	Mean	5.48 (3.84, 9.30)	4.77 (3.47, 6.90)	5.63 (3.81, 10.67)	4.76 (3.62, 6.32)	4.41 (2.46,11.15)	4.83 (3.63, 6.45)	4.58 (3.35, 5.85)
	SD	4.17 (2.44, 8.84)	4.08 (2.26, 8.05)	4.34 (2.46, 11.15)	3.32 (2.28, 5.12)	3.44 (2.13, 5.93)	3.33 (2.38, 5.27)	3.28 (2.18, 4.01)
	WAIC	217.6	214.7	219.0	127.1	125.2	128.5	NA
SI[c]	Mean	7.09 (5.07, 12.09)	6.23 (4.71, 8.63)	7.18 (5.09, 13.25)	6.27 (4.87, 8.00)	5.86 (4.63, 7.45)	6.35 (4.86, 8.07)	6.06 (4.71, 7.47)
	SD	4.14 (2.31, 9.30)	3.59 (2.05, 6.98)	4.11 (2.36, 10.96)	3.26 (2.18, 5.03)	3.12 (2.00, 5.19)	3.20 (2.29, 4.93)	3.15 (1.98, 3.90)
	WAIC	144.3	142.7	146.1	85.4	84.5	87.3	NA

NA: Not applicable
[a] Containment delay
[b] Serial interval using 26 paired data
[c] Serial interval using 17 paired data

Figure S1. Empirical distribution of serial interval with best fitted gamma, lognormal and Weibull distributions

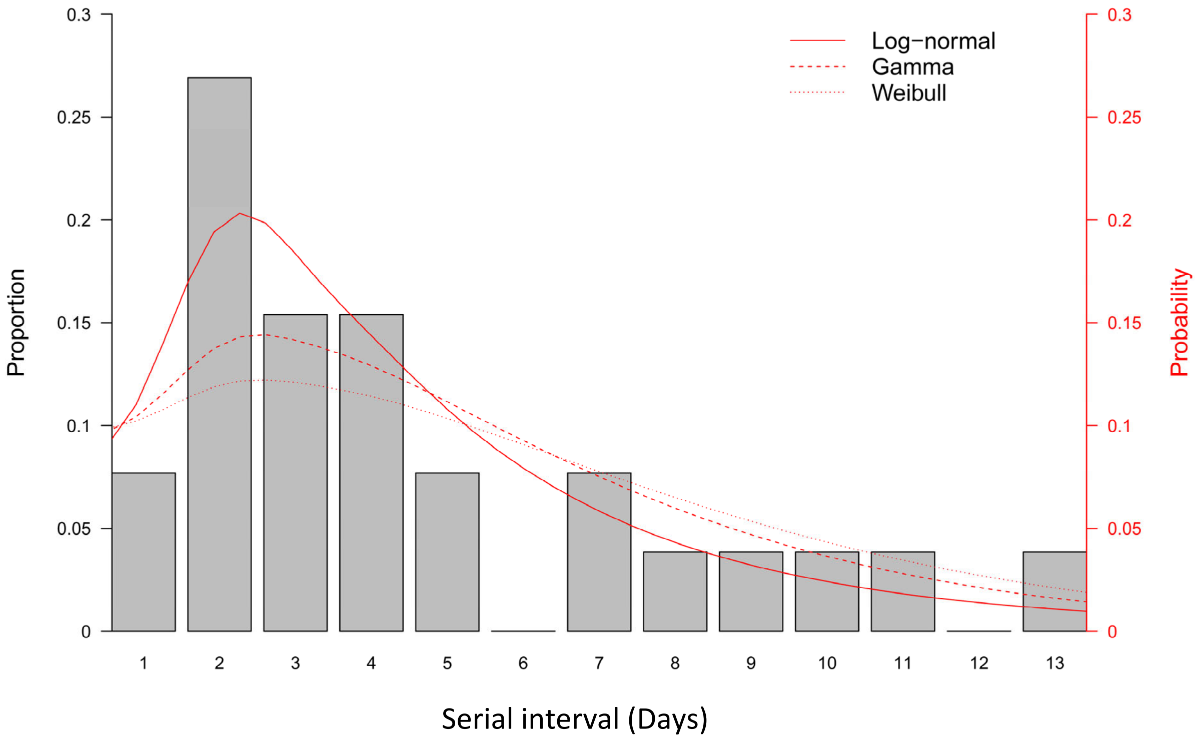

Figure S2. Epidemiological timeline from symptom onset to isolation by cluster

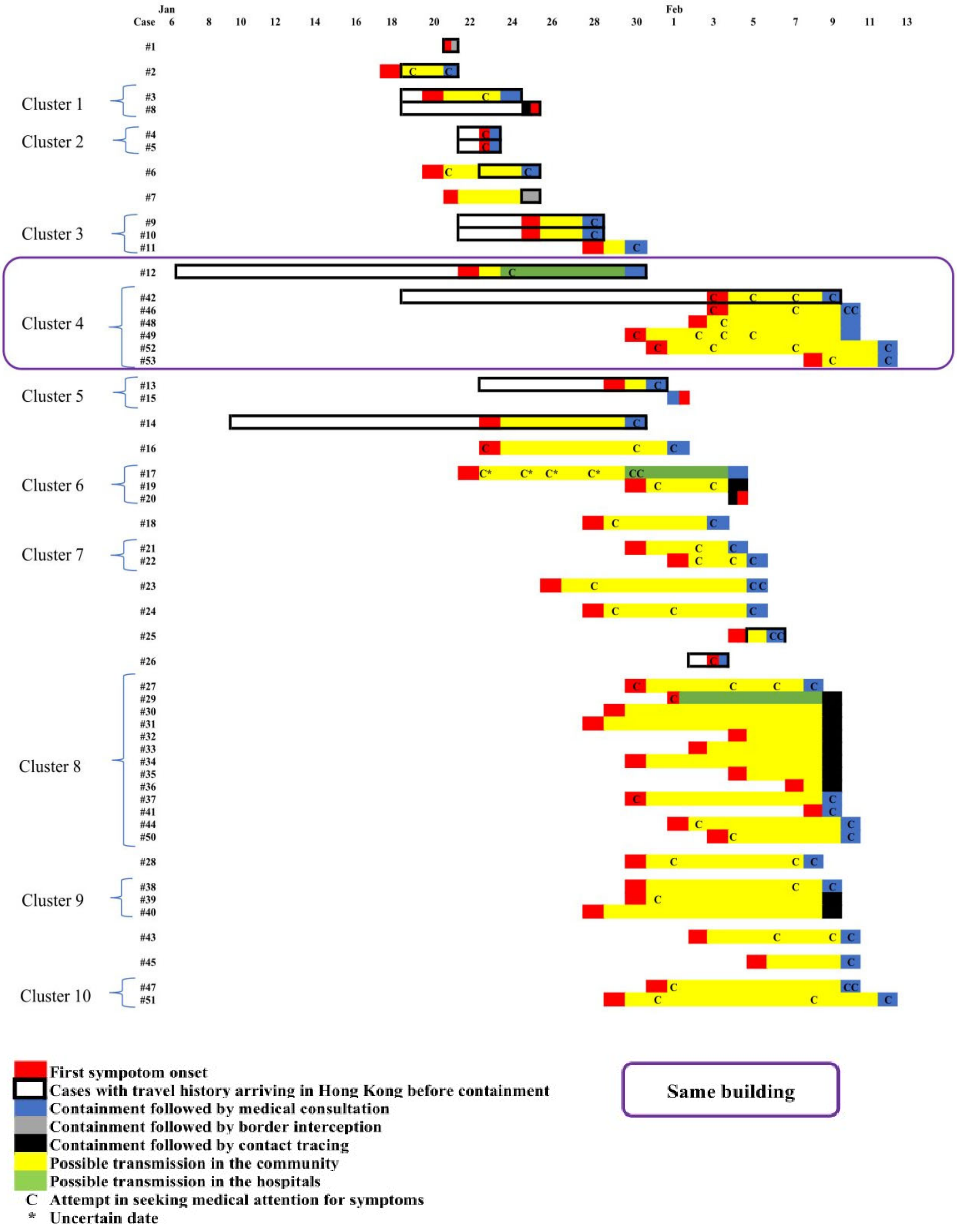

Case #30, #31, #32, #33, #34, #35, and #36 identified through contact tracing (no details in medical consultation seeking history)

Note:

1. CHP adjusted the first symptom onset of case 14 from 19 January 2020 to 23 January 2020 based on expert opinions.
2. Here are the details of the clusters:

Cluster 1: The couple were Wuhan residents who reported visiting the wet markets in Wuhan and had contact with a case in a hospital in Wuhan. The husband (case 8) was placed under quarantine and subsequently reported symptoms.

Cluster 2: The couple were Wuhan residents. They reported developing symptoms and sought medical attention and were tested positive for COVID-19.

Cluster 3: This cluster included a couple (cases 9 and 10) from Wuhan and their daughter (case 11) residing in Hong Kong. The couple developed symptoms when they were in Hong Kong. Their daughter also developed symptoms subsequently.

Cluster 4: This cluster included six cases (cases 42, 46, 48, 49, 52 and 53). They have shared food for dinner. 1. Case 12 may be linked to this cluster by living in the same building and same unit with the family (cases 42, 48 and 49).

Cluster 5: This cluster included a son travelled to Wuhan and his biological mother who resided in the same household. The mother had no travel history to Wuhan. She developed symptoms after her son was diagnosed with COVID-19.

Cluster 6: This cluster included a family of three (case 17: father; case 19: daughter; case 20: mother). They had no travel history to China. Case 17 was first diagnosed with COVID-19. Cases 19 and 20 felt ill and were diagnosed subsequently. They resided in the same household.

Cluster 7: They had travel history to China and Japan. Case 21 reported symptoms and got diagnosed with COVID-19. Case 22 was diagnosed subsequently after she had presented symptoms.

Cluster 8: This cluster included 13 cases who had no travel history during the incubation period. Cases 27, 29-37 and 41 shared food during dinner. Cases 44 and 50 did not attend the dinner. Case 44 was the colleague of case 30 while case 50 was the colleague of case 37.

Cluster 9: They resided in the same household, and had no travel history during the incubation period.

Cluster 10: They shared food during a banquet. They reported no travel history.

Figure S3. The relationship between containment delay, incubation period and (assuming positive) serial interval

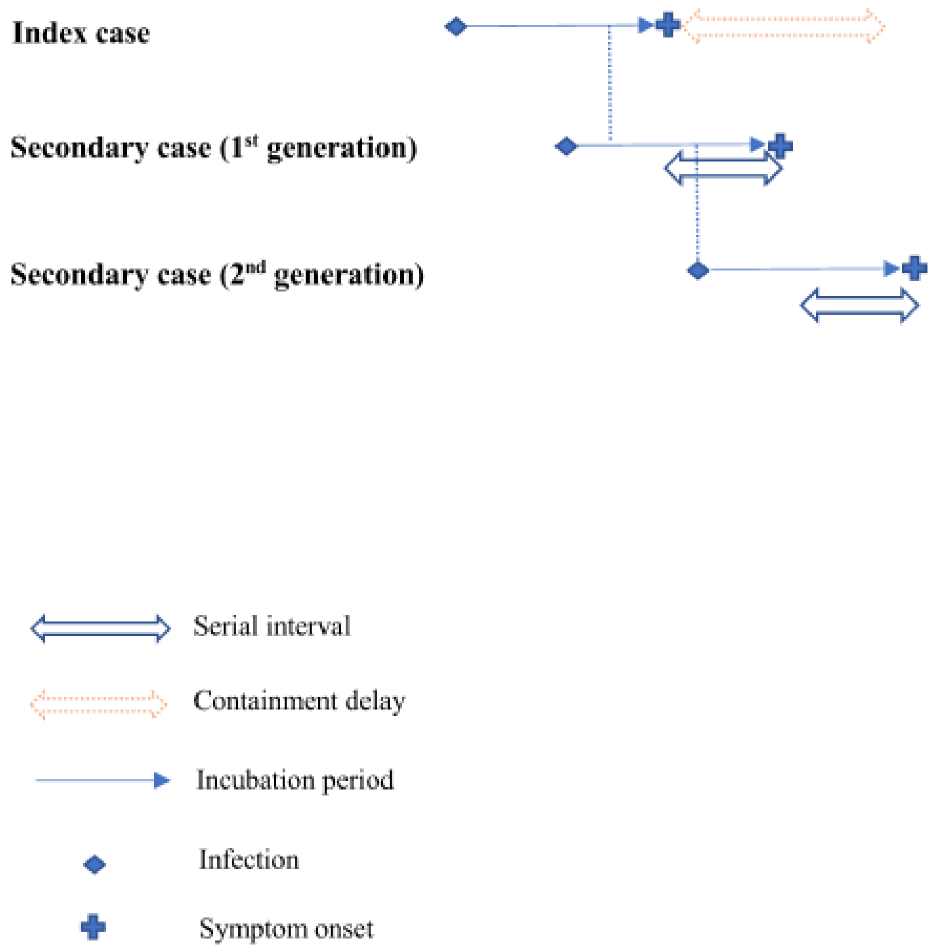

Reference

1. Vink MA, Bootsma MC, Wallinga J. Serial intervals of respiratory infectious diseases: a systematic review and analysis. Am J Epidemiol. 2014;180(9):865-75.
